# Cellulose Nanocrystal and Polymer Composite Microspheres for Methylene Blue Adsorption

**DOI:** 10.3390/polym17091205

**Published:** 2025-04-28

**Authors:** Yaxuan Deng, Zenghui Li, Rui Wang, Yue Shi

**Affiliations:** 1 School of Physical Science and Technology, Ningbo University, No. 818 Fenghua Road, Jiangbei District, Ningbo 315211, China; 2211260095@nbu.edu.cn (Y.D.); 2311690099@nbu.edu.cn (Z.L.); 2 Smart Liquid Crystal Technologies Co., Ltd., Suzhou 215500, China; rui.wang@jitri-lci.com

**Keywords:** microspheres, cellulose nanocrystals, hydrogel, methylene blue, adsorption

## Abstract

In the present study, cellulose composite microspheres were synthesized based on the reversed-phase suspension method by introducing cellulose nanocrystals (CNCs) into polyacrylamide (PAM), followed by partial hydrolysis. Their adsorption performance for methylene blue (MB) dye in aqueous solution was investigated by varying the CNC content, pH value, and particle size of the microspheres, showing excellent removal efficiency and a good regeneration performance. In addition, the adsorption kinetics were determined in accordance with the quasi-secondary kinetic model, and the equilibrium isotherm performance followed the Langmuir adsorption model. This work provides a reliable experimental basis and solid theoretical foundation for the potential application of cellulose-based composite microspheres in the field of wastewater treatment. They are expected to represent a highly efficient adsorbent material and promote the development of related fields.

## 1. Introduction

In contemporary society, the rapid development of industrial production has driven remarkable technological advancements while simultaneously imposing significant environmental burdens [1]. As a vital natural resource, water faces increasing threats from various pollutants, with industrial wastewater standing out as a primary contamination source. The discharge of substantial quantities of exhaust gases, solid wastes, and industrial effluents into aquatic systems can induce severe ecological damage [2]. Particularly concerning is dye pollution from extensive applications in textile printing, paper manufacturing, food processing, pharmaceuticals, and electroplating industries, where, for example, approximately 20,000 tons of textile dyes are released in textile industries every year [3]. These dyes demonstrate toxicity toward aquatic organisms and can undergo bioaccumulation through food chains, potentially leading to serious human health consequences [4]. For example, human exposure to MB results in health problems, including physical irritations, respiratory distress, digestive and mental disorders, abdominal disorders, and so on. When the dosage exceeds 5 mg/kg, its monoamine oxidase inhibitory property could even cause a fatal serotonin toxicity to human body [5]. Consequently, the removal of dye contaminants from water systems has become imperative.

To address this challenge, researchers have explored various wastewater treatment technologies such as electrocoagulation, chemical oxidation, photocatalysis, membrane separation, biodegradation, and adsorption [6,7,8]. As electrocoagulation requires high energy consumption, chemical oxidation demands large amounts of chemicals, photocatalysis depends on light sources and has limited efficiency, membrane separation faces issues of membrane fouling and high costs, and biodegradation is limited by the biodegradability of pollutants, adsorption has emerged as the preferred method for complex wastewater systems due to its high efficiency and versatility. This technique not only effectively removes dyes but also simultaneously eliminates multiple contaminants, including organic pollutants and heavy metal ions. With the additional advantages of operational simplicity, cost-effectiveness, and environmental compatibility, adsorption technology represents an ideal solution for diverse application scenarios [9].

Microspheres have attracted significant attention as high-performance solid adsorbents owing to their large specific surface areas [10,11,12]. Currently, a variety of materials are utilized for microsphere production, including inorganic substances such as activated carbon [13] and zeolites [14], as well as organic counterparts like polymers [15] and resins [12]. These materials present distinct advantages and limitations in terms of adsorption performance, structural stability, and cost-effectiveness. Cellulose, as the most abundant natural polymer, offers an ideal matrix for adsorbent development due to its renewable nature, biodegradability, non-toxicity, low cost, and excellent biocompatibility [16,17]. Particularly noteworthy are cellulose nanocrystals (CNCs), rod-shaped crystalline domains obtained through sulfuric acid hydrolysis, which exhibit unique properties including nanoscale effects, a high specific surface area, and exceptional mechanical strength [18,19]. These characteristics endow CNCs with broad application prospects in biomedical engineering, environmental protection, energy storage, and composite reinforcement [20,21,22], while demonstrating remarkable potential for dye adsorption [23,24]. Significant progress has been made in developing CNC-based composite microspheres. For instance, Li et al. successfully prepared porous CNC/MnO_2_/sodium alginate (SA) microspheres via the freeze-drying templated emulsion method, reporting an improved adsorption performance [25]. Zhou et al. developed CNC-reinforced hydrogels with enhanced cationic dye adsorption capacity and investigated their adsorption mechanisms [26].

This study incorporated CNCs into anionic polyacrylamide (APAM) matrices, applying reversed-phase suspension method for composite microsphere preparation. APAM matrices inherently possess a high swelling capacity and tunable functional groups on their surface, making them particularly effective for ionic contaminant capture [26,27,28]. However, traditional APAM systems suffer from poor regeneration efficiency and inadequate structural stability. The incorporation of nanoscale CNC rods not only enriches the porous structure of the microspheres but also leads to dual networks incorporating hydrogen bonds and chemical bonds, achieving a significant improvement in MB adsorption capacity with good regenerability. The adsorption mechanism was investigated in kinetic, isothermal, and thermodynamic aspects, providing both a reliable experimental basis and a solid theoretical foundation for the potential application of cellulose-based composite microspheres in the field of wastewater treatment.

## 2. Materials and Methods

### 2.1. Reagents

CNCs were purchased from ScienceK Co., Ltd. (Huzhou, China), with a length of around 200 nm, a diameter of approximately 10 nm, and a zeta potential of −48 mV. Acrylamide (AM; electrophoresis grade; ≥99%) was obtained from J&K Scientific Co., Ltd. (Shijiazhuang, China). Irgacure 2959 (photoinitiator; ≥98.0%; HPLC), *N*,*N*’-methylenebisacrylamide (Bis; crosslinker; ≥99%), petroleum ether (AR, bp 60–90 °C), light liquid paraffin (density: 0.84–0.86), Span 80 (surfactant; viscosity 1000–2000 mPa·s (20 °C)), and glutaraldehyde (GA; 50%) were all purchased from Aladdin (Shanghai, China). Caustic soda (NaOH; granular; AR), hydrochloric acid (HCl; 500 mL; ≥37%), sodium chloride (NaCl; AR), and methylene blue (MB, ≥95%) were obtained from Sinopharm Chemical Reagent Co., Ltd. (Shanghai, China) and Shanghai Titan Scientific Co., Ltd. (Shanghai, China), respectively. All reagents were used without further purification.

### 2.2. Synthesis

**Figure 1:**
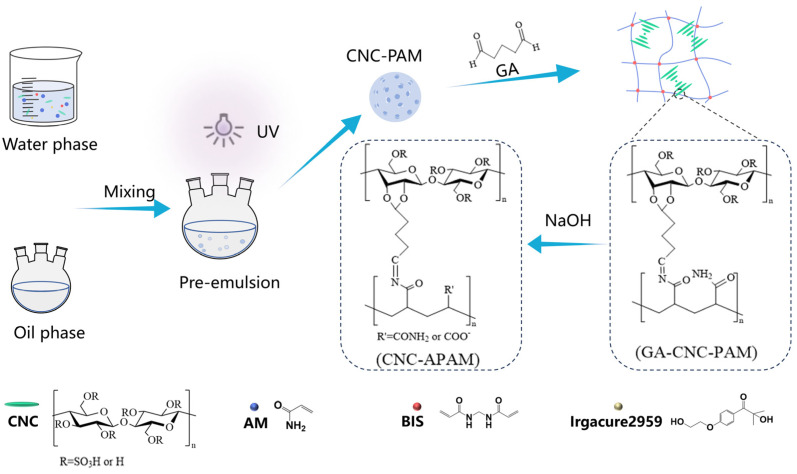
Schematic diagram of the preparation process of composite microspheres.

The preliminary composite microspheres (denoted as CNC-PAM) were prepared via the reverse-phase suspension method, as shown in Figure 1. Taking the CNC40-PAM60 microspheres as an example, 6 g of CNCs were weighed and homogeneously dispersed in 100 mL of deionized water using an ultrasonic cell crusher (Scientz Y92-IIN, Ningbo, China). Subsequently, 10 g of AM, 0.33 g of Bis, and 0.33 g of Irgacure 2959 were added and stirred thoroughly. The resulting suspension was then introduced into 500 mL of liquid paraffin containing 10 wt% Span 80, followed by mechanical stirring at 400 rpm for 4 min (Xuanwo HD2010W, Shanghai, China). Upon the completion of the reaction, the suspension was cured under a 365 nm LED UV lamp (Tiandou Lighting, Zhongshan, China) for 1 h using a transparent glass lid. The microspheres were subsequently washed sequentially with petroleum ether, ethanol, and deionized water via centrifugation (Xiangyi Instruments H1750R, Changsha, China) to obtain CNC40-PAM60 microspheres. Finally, 10 mL of 5% GA solution was added to 10 g of swollen microspheres, and the mixture was stirred at 30 °C for 2 h to yield secondary cross-linked composite microspheres (denoted as GA-CNC-PAM).

Subsequently, anionic composite microspheres (denoted as CNC-APAM) were prepared via alkaline hydrolysis with the hydrolysis degree set at 30%. A 30% hydrolysis degree provides sufficient carboxyl groups to enhance the electrostatic adsorption capacity of the microspheres for dye molecules while maintaining their structural stability. Excessive hydrolysis may lead to structural damage to the microspheres, thereby affecting their adsorption performance [26,27,28]. The alkali dosage was calculated using the following formula:

*m*_NaOH_ = 30% *m*_AM_ *M*_NaOH_*/M*_AM_, (1)

where *M*_AM_ and *M*_NaOH_ are the molar masses of AM and NaOH, and *m*_AM_ and *m*_NaOH_ are the masses of AM and NaOH, respectively. Here, 1.0129 g of NaOH was dissolved in 594 mL of deionized water, and 10 g of pre-swollen GA-CNC-PAM microspheres was added, thoroughly mixed, and transferred into a water bath shaker (Shenghua Instrument, SHA-B, Suzhou, China) maintained at 60 °C for 5 h. At the end of the reaction, the product was washed with deionized water to a neutral state. Composite microspheres with CNC solid contents of 0 wt%, 10 wt%, 20 wt%, 30 wt%, 40 wt%, and 50 wt% were prepared, designated as CNC0-APAM100, CNC10-APAM90, CNC20-APAM80, CNC30-APAM70, CNC40-APAM60, and CNC50-APAM50, respectively. Microspheres of different sizes were obtained by adjusting the stirring speed followed by sieving through standard mesh sieves (60–65, 100–120, 200–280, and 400–500 mesh, Shanghuafeng Hardware & Instrument Co., Ltd., Shaoxing, China).

### 2.3. Characterization

Infrared spectra were measured using a Fourier Transform Infrared Spectrometer (FT-IR, ThermoFisher iS50, Waltham, MA, USA). The absorbance spectra were measured using a UV–visible spectrophotometer (Shimadzu UV2600, Kyoto, Japan). Particle size distributions were determined via a laser particle size analyzer (Beckman Coulter LS13320, Brea, CA, USA). The microstructure of the composite microspheres was examined under a polarized optical microscope (POM, Nikon LV100N POL, Tokyo, Japan). For scanning electron microscopy observation (SEM, Zeiss Gemini 300, Oberkochen, Germany), the water-containing composite microspheres were dried with a freeze dryer (Boyikang PiLot1-2LD, Beijing, China) and sprayed with 5 nm Pt (Quorum Q150TES, Laughton, UK) before characterization. To observe the internal porous structure, gel blocks with identical compositions were freeze-dried and cryogenically fractured for cross-sectional SEM analysis. The correlation between the particle size and specific surface area was investigated by the nuclear magnetic resonance spectrometer (NMR, Magritek Spinsolve 60 Phosphorus, Aachen, Germany), where the spin–spin relaxation time was determined using a Carr–Purcell–Meiboom–Gill (CPMG) sequence with a 0.5 ms pulse interval.

Swelling ratio measurements were conducted using gel blocks instead of microspheres to improve operational feasibility. First, a gel block with the initial mass (*W*_1_) was immersed in deionized water. After 24 h, the swollen gel block was carefully removed with tweezers, and residual surface liquid was blotted using filter paper. The swollen mass (*W*_2_) was immediately recorded, and the swelling ratio (*SR*, g·g^−1^) was calculated using the following equation:

*SR* = (*W*_2_ − *W*_1_)/*W*_1_, (2)

### 2.4. Adsorption Investigation

All experimental data were averaged from three independent replicates. To evaluate the adsorption performance of composite microspheres, 2.8 mL of swollen microspheres was transferred into 25 mL of MB solution and incubated in a 30 °C water bath shaker for varying adsorption durations. As the adsorption process proceeded, the MB molecules were gradually captured by the microspheres, resulting in a corresponding decrease in solution concentration and light absorbance. Subsequently, the supernatant absorbance was measured at MB’s characteristic absorbance wavelength (664 nm) using a pre-calibrated standard curve. The adsorption capacity (*q*_t_, mg/g) at different time points was calculated as follows:

*q*_t_ = (*C*_0_ − *C*_t_) *V*/*m*, (3)

where *C*_0_ (mg/L) is the initial concentration of MB; *C*_t_ (mg/L) is the concentration of MB in the supernatant after measuring the absorbance at the different time t; *V* (L) is the volume of the MB solution; and *m* (g) is the mass of the composite microspheres. When reaching equilibrium, where 2 h was applied, *C*_t_ reached *C*_e_ and *q*_t_ reached *q*_e_. The removal efficiency (*R*_e_, %) is calculated using the following formula:

*R*_e_ = (*C*_0_ − *C*_e_) 100/*C*_0_, (4)

To conduct adsorption experiments under varying pH conditions, the pH value was adjusted to 1, 3, 5, 7, 9, and 11 using 0.1 M HCl and 0.1 M NaOH solutions. To investigate the effect of ionic strength, parallel adsorption tests were performed in 0–0.4 M NaCl solutions at optimal pH condition. To evaluate the reusability of the composite microspheres, MB-loaded composite microspheres were mixed with 50 mL of 0.1 M HCl to completely elute adsorbed MB, followed by washing with deionized water until a neutral pH was achieved for subsequent reuse tests.

To investigate the adsorption isotherm of the composite microspheres, the MB solutions were prepared at concentrations of 100, 130, 160, 190, 220, 250, 280, and 310 mg/L. Then, 25 mL of each solution was added to a centrifuge tube containing 2.8 mL of swollen CNC40-APAM60 composite microspheres (0.01 g), followed by incubation in a temperature-controlled shaker (Shenghua Instrument, SHA-B, Suzhou, China) at 30 °C, 40 °C, 50 °C, and 60 °C for 2 h to achieve adsorption equilibrium.

## 3. Results and Discussion

### 3.1. Characterization of Composite Microspheres

**Figure 2:**
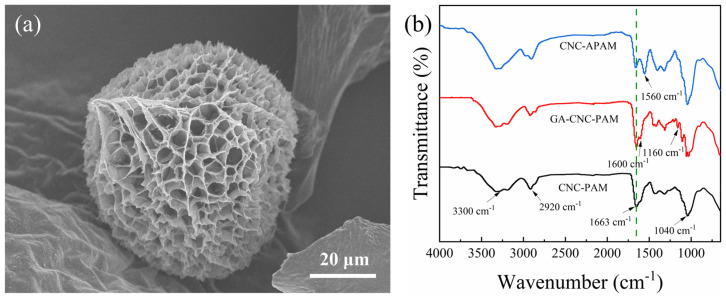
(**a**) SEM image of a CNC40-PAM60 microsphere; (**b**) FT-IR spectra of CNC-PAM, GA-CNC-PAM, and CNC-APAM composite microspheres.

The SEM micrograph of a composite microsphere reveals a distinct porous structure, as shown in Figure 2a.

FT-IR spectra of CNC-PAM, GA-CNC-PAM, and CNC-APAM microspheres are presented in Figure 2b. In the spectrum, the broad absorbance band near 3300 cm^−1^ is assigned to the stretching vibrations of -OH and -NH groups. The peak around 1040 cm^−1^ is attributed to the stretching vibration of C-O-C groups in CNC. The characteristic absorbance peak near 2920 cm^−1^ corresponds to the stretching vibration of -CH groups. Additionally, the peak at 1663 cm^−1^ is attributed to the C=O on the amide group of the PAM in the CNC-PAM spectrum. For GA-CNC-PAM, an absorbance band emerges at 1600 cm^−1^, which was attributed to the reaction between the -NH_2_ group of PAM and the -CHO group of GA [29]. In the CNC-APAM spectrum, a peak at 1560 cm^−1^ appears, which originates from the asymmetric stretching vibration of -COO^−^ [30], while the intensity of the absorbance peak originating from C=O on the amide group at 1663 cm^−1^ is obviously weakened, indicating the partial hydrolysis of -CONH_2_ groups to -COO^−^ during alkaline treatment.

### 3.2. Adsorption Behavior of CNC-APAM Composite Microspheres

#### 3.2.1. Effect of pH and Ionic Strength

**Figure 3:**
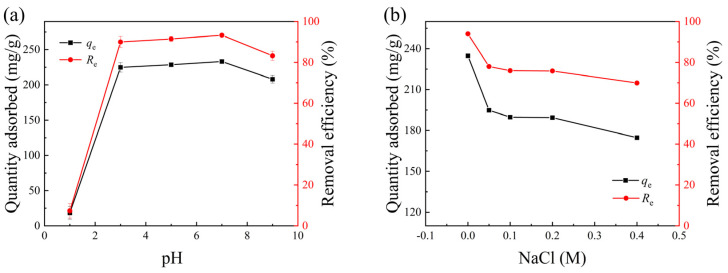
(**a**) Effect of pH and (**b**) effect of NaCl concentration on the MB adsorption by CNC40-APMA60 microspheres at equilibrium.

pH value usually affects the adsorption performance of ionic dyes [31,32,33]. As shown in Figure 3a, the adsorption capacity of CNC-APAM composites for MB exhibited an initial increase followed by a decrease as the pH rose from 1 to 9. The maximum adsorption capacity is 233.18 mg/g, appearing at pH = 7. At lower-pH conditions, the presence of a large amount of H^+^ in the solution competed with the cations of MB for adsorption, resulting in a decrease in the adsorption rate of the composite microspheres on MB dye [32]. With increasing pH, the negative charges on the surface increased and attracted MB cations more easily. However, at a pH above 7, the OH^−^ in the solution may affect the electrostatic attraction between -COO^−^ on the microspheres and the positively charged MB dyes, leading to a diminished adsorption capacity [33]. In our experiment, the MB solution lost its maximum absorbance peak at 664 nm when the pH was 11, probably due to the reduction reaction of MB in a highly alkaline environment. Therefore, pH = 7 was approved as the optimum adsorption environment, and the following tests were conducted at this value.

Ionic strength is also a critical factor influencing the performance of ionic dye adsorbents. Therefore, NaCl solutions with concentrations ranging from 0 to 0.4 M were selected to evaluate the interference effects of ionic strength on MB molecule adsorption sites, with the experimental results shown in Figure 3b. The data revealed that when the NaCl concentration increased from 0 to 0.4 M, the removal efficiency decreased, which was attributed to intensified competitive adsorption between Na^+^ ions and MB molecules for active sites [34]. Therefore, the following tests were conducted without ionic addition to explore the adsorption performance of CNC40-APMA60 microspheres.

#### 3.2.2. Effect of CNC Concentration

**Figure 4:**
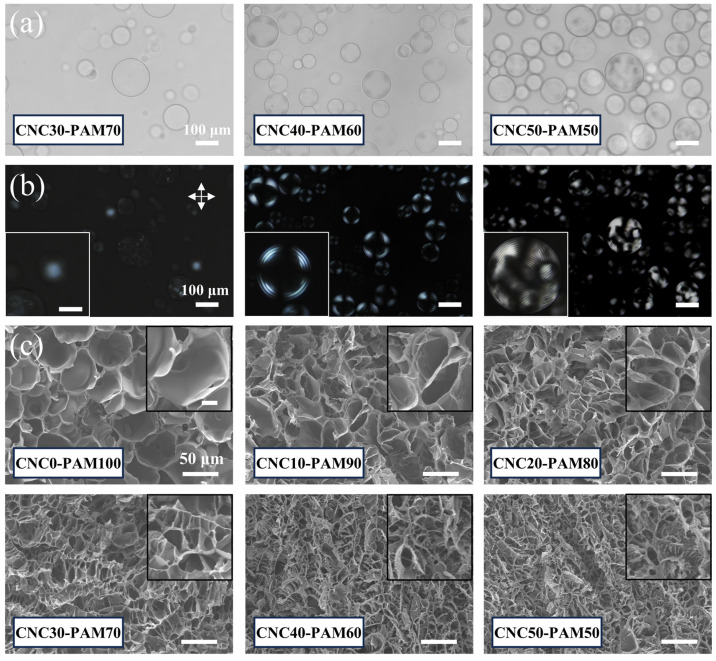
(**a**) Microscopic images and (**b**) POM images of CNC-PAM microspheres with different contents. The crossed arrows indicate crossed polarizers, and the scale bar in the insets is 50 μm. (**c**) Cross-sectional SEM images of the gel blocks of CNC-PAM with different contents. The scale bar in the inset is 10 μm.

The CNC-PAM composite microspheres with varying CNC contents were observed with a microscope as shown in Figure 4a,b. As the CNC content increases, the fingerprint textures become more distinct and regular under POM, attributed to the formation of cholesteric liquid crystal phase when CNC suspension reaches critical concentration [35]. The internal porous structures of the composite material with different CNC contents were observed by SEM (Figure 4c), revealing a progressive reduction in pore size and a heterogeneous microstructure with increasing CNC content. This structural evolution originates firstly from extensive hydrogen bonding between hydroxyl groups on CNCs and amino groups along PAM chains promoting cross-linking densification, yielding a more compact polymeric matrix. Furthermore, increasing CNC content induces periodic helical structures in the matrix due to the cholesteric liquid crystal organization, and more rod-like micro/nanoscale CNC fibers appear on the pore surfaces to enhance the surface roughness. This clearly demonstrates that the CNC content in the composite material significantly affects the microstructure of the composite spheres.

The average size of the microspheres was approximately 62 µm regardless of CNC content, with reasonable deviation as listed in Table 1. The removal efficiency of MB initially increased dramatically with CNC content and then slightly decreased. This is because the anionic groups in the whole composite microsphere system increase with the addition of CNC, where the sulfonic ester groups on the CNCs provide more adsorption sites for the MB cations to attach to, strengthening the adsorption of MB through electrostatic attraction. At the same time, more rod-shaped CNCs construct more complex pore and channel structures in the composite microspheres, which enlarge the specific surface area and allow more MB molecules to reach the adsorption sites. However, when the CNC concentration further increases, the interactions between MB and CNCs compete with the adsorption sites of anion groups on APAM [36]. And the more tightly packed and heterogeneous internal structure could also restrict the entry of water molecules, illustrated by the *SR* value in Table 1, probably limiting the access of MB molecules to the interior adsorption sites of the microspheres and thus restricting the removal efficiency. Here, the CNC40-APAM60 microspheres were selected for the subsequent experiments. On the one hand, the CNC40-APAM60 microspheres exhibit a good efficiency of 93.4%, and their adsorption efficiency is the highest compared with other combinations. On the other hand, its low swelling rate is beneficial for the reuse rate of the material.

#### 3.2.3. Effect of Microsphere Size

**Figure 5:**
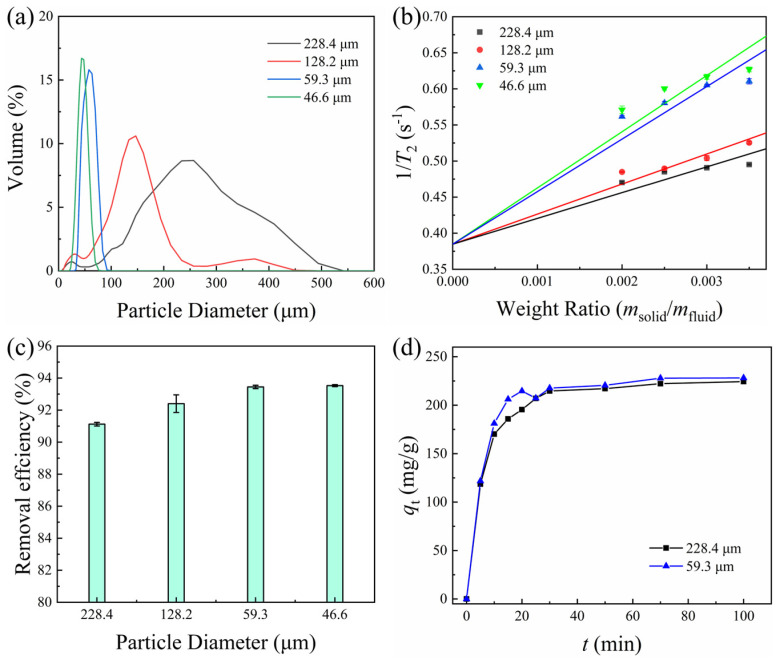
(**a**) Particle size distribution of CNC40-APAM60 composite microspheres; (**b**) plot of relaxation rate versus weight ratio of different particle sizes; (**c**) effect of particle size on the adsorption of MB; (**d**) effect of particle size on adsorption kinetic profile of MB.

Adsorbent materials with different particle sizes often show significant differences in adsorption performance since particle size directly affects specific surface area. In this study, CNC40-APAM60 composite microspheres with different particle sizes were prepared, as shown in Figure 5a. The average particle sizes were 228.4, 128.2, 59.3, and 46.6 µm, respectively.

To reveal the intrinsic correlation between the specific surface area and the particle size of composite microspheres, the low-field NMR technique was employed in this study for an in-depth investigation. The correspondence between the relaxation rate (1/*T*_2_) and the specific surface area was established by a rigorous mathematical model [37]:

1/*T*_2_ = 1/*T*_2b_ + (*ρ*_2_*SSA*_solid_)·(*m*_solid_/*m*_fluid_), (5)

where *T*_2_ is the transverse relaxation time, *T*_2b_ is the spin–spin relaxation time constant of the bulk fluid, *SSA*_solid_ is the wet specific surface area, and *m*_solid_/*m*_fluid_ is the mass ratio of the composite microspheres to water. *ρ*_2_ is the surface relaxivity, which characterizes the efficiency of surface-enhanced relaxation. Given that all samples in this study have the same conditions except for particle size, it is reasonable to assume that they have the same *ρ*_2_, and the slope is proportional to the specific surface area of *SSA*_solid_. The underlying mechanism is that the larger specific surface area results in a significant increase in the frequency of contact between the surface atoms and the solvent molecules, leading to a reduction in the transverse relaxation time [38].

The relationship between the relaxation rate and weight ratio was measured for the CNC40-APAM60 aqueous suspension, as shown in Figure 5b. It was clearly observed that the smaller microspheres had shorter relaxation times. Linear fitting according to Equation (5) showed that the slope increased as the particle size of the composite microspheres decreased, indicating a larger specific surface area. The adsorption performance of MB on microspheres with different particle sizes was further investigated, as shown in Figure 5c,d. Both increased removal efficiency and a shortened equilibrium adsorption time were observed for smaller microspheres. This is attributed to the fact that the larger specific surface areas provide more adsorption sites, while the porous microstructure remains the same with identical compositional conditions, therefore increasing the adsorption capacity of dyes [39,40].

#### 3.2.4. Reusability

**Figure 6:**
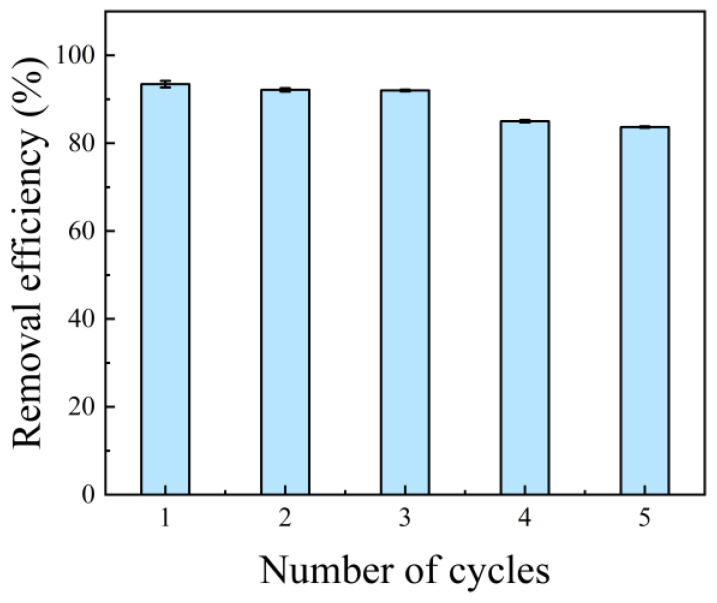
Reusability of CNC40-APAM60 composite microspheres for MB removal in 5 cycles.

Reusability is one of the important indices used to evaluate the feasibility of adsorbents. As shown in Figure 6, CNC40-APAM60 could recover up to 90% of its original adsorption capacity after five cycles, which is good enough compared to the performance of other related studies [40,41,42]. This fully demonstrates its good desorption capacity and indicates that CNC-APAM composites have good potential and development prospects in the field of adsorbent applications.

### 3.3. Adsorption Mechanism

#### 3.3.1. Adsorption Kinetics

**Figure 7:**
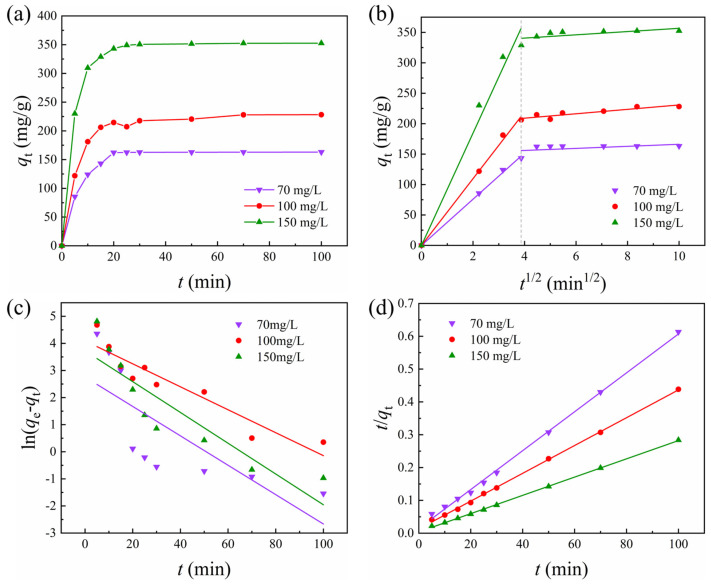
(**a**) Adsorption kinetic profile of MB on CNC40-APAM60 microspheres. (**b**) Intraparticle diffusion model of the adsorption process. (**c**) Pseudo-first-order model of the adsorption process. (**d**) Pseudo-second-order model of the adsorption process.

Adsorption kinetics is a vital methodological approach to elucidating the adsorption mechanism. It can determine the rate of adsorption, predict adsorption behavior, and help in understanding the interactions between adsorbents and adsorbates. As shown in Figure 7a, the adsorption kinetics of MB by CNC40-APAM60 composite microspheres was investigated with varying MB concentration from 70 mg/L to 150 mg/L. All of them exhibited a rapid increase during the initial adsorption phase and then gradually slowed down and reached equilibrium. In the initial phase, the surface of the composite microspheres contains a large number of carboxyl groups and sulfate ester groups. These negatively charged groups interact with the cations of the MB dyes through electrostatic attraction, resulting in the rapid adsorption of the cationic dye [43]. As the number of available adsorption sites decreases, the adsorption rate slows down and eventually reaches saturation. Different initial MB concentrations gave different adsorption amounts of MB, but with a linear dependence, as expected.

An in-depth investigation into the adsorption mechanism of CNC-APAM composite microspheres on MB was carried out using the intraparticle diffusion models [44],

*q*_t_ = *K*_id_ *t*^1/2^, (6)

*q*_t_ = *K*_id_ *t*^1/2^+*C*, (7)

where *K*_id_ (mg·g^−1^·min^−1/2^) is the rate constant for the intraparticle diffusion model, and *C* is a constant related to the extent of the boundary layer effect. As shown in Figure 7b, the adsorption curve clearly shows two significant regions. The fitting results are summarized in Table 2. The slope value decreases significantly from the first region to the second region, indicating that the removal rate of MB in solution decreases gradually over time. In the first region, the zero intercept indicates the intraparticle diffusion process where the MB molecules firstly diffuse into the porous structure of the adsorbent and finally reach an equilibrium state. In the second region, the non-zero *C*-value implies that the equilibrium of intraparticle diffusion is disrupted due to the adsorption of MB onto the active sites of the adsorbent [45,46]. Here, the adsorption kinetics of different initial MB concentration has the same turning point, indicating that the adsorption mechanism of CNC-APAM composite microspheres does not depends on the initial MB concentration in our studied range.

Pseudo-first-order [47] and pseudo-second-order [41] models were further applied to investigate the adsorption mechanism of microspheres:

ln(*q*_e_ − *q*_t_) = ln*q*_e_ − *K*_1_*t*, (8)

*t/q*_t_ = 1/*K*_2_*q*_e_^2^ + *t/q*_e_, (9)

where *q*_e_ (mg/g) is the amount of MB adsorbed at equilibrium, *K*_1_ (min^−1^) and *K*_2_ (g/mg·min) are the equilibrium rate constants for the pseudo-first-order and the pseudo-second-order models, respectively. Figure 7c,d show the two fitting kinetic curves, and the specific parameters are listed in Table 2. It can be clearly seen that the kinetic behavior of MB adsorption by the composite microspheres is better characterized by the pseudo-second-order kinetic model, indicating that this adsorption procedure involves both diffusion and surface adsorption processes [48].

**Table 2:** Kinetic parameters of MB adsorption by CNC40-APAM60 composite microspheres.

Models	Kinetic Parameters	70 mg/L	100 mg/L	150 mg/L
Intraparticle diffusion	*K*_1d_ (mg·g^−1^·min^−1/2^)	37.9286	54.7863	92.2263
	*R* ^2^	0.9993	0.9989	0.9934
	*K*_2d_ (mg·g^−1^·min^−1/2^)	1.6916	3.6212	2.6779
	*C* (mg/g)	149.2161	194.5331	329.9028
	*R* ^2^	0.2632	0.8365	0.4912
Pseudo-first-order	*K*_1_ (min^−1^)	0.0542	0.0424	0.0568
	*q*_e_ (mg/g)	15.7615	59.9098	41.4302
	*R* ^2^	0.5865	0.8832	0.8088
Pseudo-second-order	*K*_2_ (g/mg·min)	0.0024	0.0014	0.0020
	*q*_e_ (mg/g)	168.6341	235.8491	359.7122
	*R* ^2^	0.9981	0.9993	0.9996

#### 3.3.2. Adsorption Isotherm

**Figure 8:**
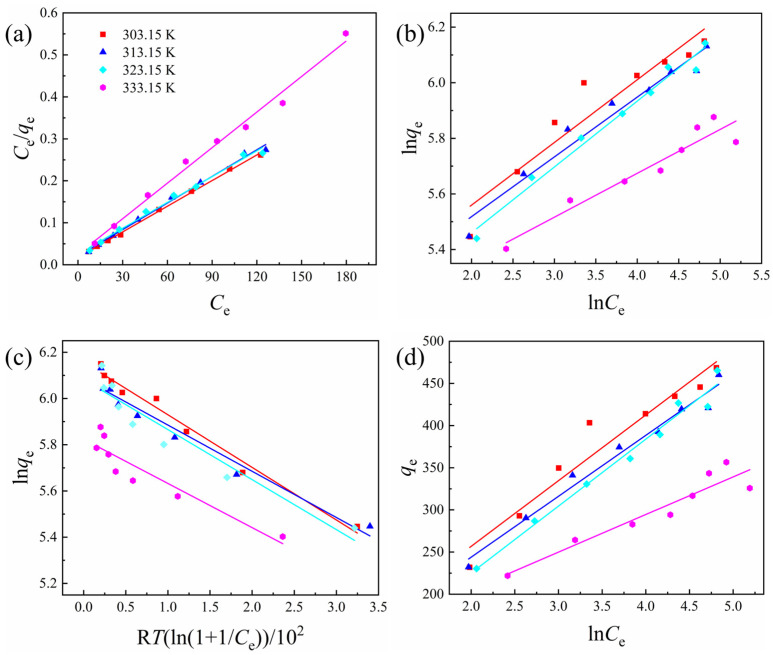
Comparison of adsorption isotherm models of MB by CNC40-APAM60 composite microspheres at different temperatures: (**a**) Linearized Langmuir, (**b**) Freundlich, (**c**) D-R, and (**d**) Temkin isotherm models.

In order to study the adsorption mechanism and evaluate the adsorption capacity of CNC-APAM microspheres, four adsorption isotherm models, Langmuir, Freundlich, Dubinin–Radushkevich (D-R), and Temkin, were selected to fit the experimental data obtained at different temperatures, as shown in Figure 8. The specific parameters of these isotherm models are provided in Table 3.

The Langmuir adsorption isotherm model describes the adsorption process in a monomolecular layer, assuming that the adsorbent surface is homogeneous and the adsorbed molecules do not interact with each other [49,50]. The model can be expressed as follows:

*C*_e_/*q*_e_ = *C*_e_/*q*_m_ + 1/*q*_m_*K*_L_, (10)

where *C*_e_ (mg/L) is the equilibrium concentration of the dye solution, *q*_e_ (mg/g) is the equilibrium adsorption capacity of MB, *q*_m_ (mg/g) is the maximum adsorption capacity of MB, and *K*_L_ is the Langmuir isothermal adsorption constant.

The Freundlich adsorption isotherm model applies to the multilayer adsorption behavior of adsorbates on the adsorbent surfaces [51,52], making it suitable for a wider range of adsorption systems. The Freundlich model is expressed as follows:

ln*q*_e_ = ln*K*_F_ + ln*C*_e_/*n*, (11)

where *K*_F_ (L/g) is the Freundlich constant reflecting the adsorption capacity, and *n* is the model exponent. The term 1/*n* corresponds to the inhomogeneity of the adsorbed surface.

The D-R model is based on the assumption that a Gaussian adsorption potential exists between the adsorbent surface and the adsorbed molecules [53,54]. This model is typically used to describe the adsorption of water vapor and gases by microporous adsorbents. The D-R isotherm can be linearized in the following form:

ln*q*_e_ = ln*q*_m_ − *K*_D_*ε*^2^, (12)

where *K*_D_ is the D-R isothermal adsorption constant. The formula for *ε* is *ε* = R*T*ln(1 + 1/*C*_e_), where R (8.314 J/mol·K) is the ideal gas constant and *T* is the absolute temperature.

The Temkin adsorption isotherm model is characterized by the presence of interactions between adsorption sites [55]. The linear form of the model is expressed as follows:

*q*_e_ = R*T*ln*A*/*B* + R*T*ln*C*_e_/*B*, (13)

where *B* is the Temkin model constant, and *A* is related to the maximum binding energy of the adsorbent, reflecting the affinity of the adsorbent for the adsorbate. The larger the value of *A*, the greater the affinity between the adsorbent and the adsorbate.

As shown in Figure 8 and Table 3, the Langmuir isotherm model provides a better fit, with the coefficient of determination (*R*^2^) value closer to one. This indicates that the cationic dye MB forms a monolayer on the internal surfaces of CNC-APAM microspheres, promoting the adsorption process [56]. According to the Langmuir isotherm model, the maximum adsorption capacity of the composite microspheres was 490.12 mg/g at 303.15 K, decreasing to 354.61 mg/g at 333.15 K. The values of *q*_m_ and *K*_L_ show a decreasing trend as the temperature increased, indicating that the adsorption process is exothermic, with the system releasing heat to the outside [42]. Additionally, the loose and porous structure of the CNC-APAM composite microspheres is not preferred as temperature increases, which may also contribute to the decrease in *q*_e_ at higher temperatures [57].

#### 3.3.3. Adsorption Thermodynamics

Thermodynamic analyses were conducted to assess the feasibility of the adsorption process from an energetic perspective. By calculating the thermodynamic parameters of Gibbs free energy change (Δ*G*), enthalpy change (Δ*H*), and entropy change (Δ*S*), we can determine whether the adsorption processes spontaneously and identify the driving force for adsorption. These three parameters are determined using the following equations [58]:

*K*_d_ = *q*_e_/*C*_e_, (14)

∆*G* = −R*T*ln*K*_d_, (15)

ln*K*_d_ = −∆*H*/R*T* + ∆*S/*R, (16)

where *K*_d_ is the Langmuir constant.

Δ*H* and Δ*S* were calculated using the slope and intercept of ln*K*_d_ plotted against 1/*T*. The specific parameters are summarized in Table 4. The negative Δ*G* is values in the studied temperature range indicate spontaneous adsorption interaction between MB and CNC-APAM microspheres. The negative Δ*H* value reveals the exothermic nature of the adsorption process. The absolute value of Δ*H* is less than 84.00 kJ/mol, indicating that the adsorption of MB on CNC-APAM is mainly physisorption [59]. The negative Δ*S* < 0 value suggests that the solid/liquid interface becomes less random with more adsorbed MB molecules, and the adsorption process is enthalpy-controlled.

#### 3.3.4. Adsorption Mechanism and Comparison

The adsorption mechanism of MB onto CNC-APAM microspheres was thoroughly explored using kinetic, isothermal, thermodynamic analyses. Kinetic studies revealed a two-phase adsorption procedure including intraparticle diffusion and adsorption processes, which was further confirmed by the pseudo-second-order kinetic model. The adsorption isothermal analysis gave a better fit with the Langmuir isotherm model, indicating a monolayer adsorption process. The decrease in maximum adsorption capacity (*q*_m_) and Langmuir constant (*K*_L_) with temperature suggests exothermic adsorption. Thermodynamic parameters have shown that the adsorption was spontaneous (Δ*G* < 0) and exothermic (Δ*H* < 0). The absolute value of Δ*H* being less than 84.00 kJ/mol indicates that physical adsorption is predominant, probably through electrostatic attraction. In summary, the heterogeneous porous structure in microspheres provides diffusion pathways and adsorption sites for MB molecules, enabling monolayer adsorption dominated by electrostatic attraction. This mechanistic understanding supports the use of CNC-APAM composite microspheres in wastewater treatment applications.

A comparative analysis of the maximum adsorption capacity obtained from Langmuir isotherm fitting with previously reported adsorbents is listed in Table 5. The CNC-APAM composite microspheres developed in this work demonstrate superior MB adsorption capacity compared to most other materials. In Ref. [60], carboxymethyl cellulose/organo-montmorillonite (CCMC/OMMT) composite hydrogels had a similar maximum adsorption capacity. Both enriched anionic groups and a rough, irregular surface morphology were reported, which was probably the reason for the comparable maximum adsorption capacities. Therefore, both the heterogeneous porous structure and the abundant anionic functional groups in the CNC-APAM composite microspheres synergistically contribute to their superior adsorption capacity in cationic dye removal applications.

## 4. Conclusions

In this paper, CNC-APAM composite microspheres were synthesized, and their adsorption properties for MB in aqueous solution were investigated using the batch equilibrium method. The adsorption amount of MB was found to be closely related to the initial pH of the solution, the specific surface area, and the CNC content of the microspheres, which could be applied to control the internal porous structures. The maximum removal efficiency (*R*_e_) of MB by CNC-APAM could reach 93.441% with a good desorption capacity, demonstrating its great potential as a highly efficient and reusable adsorbent. Further analysis revealed that the adsorption of MB on CNC-APAM composite microspheres followed the pseudo-second-order kinetic model and the Langmuir adsorption model. Isothermal studies indicated that the adsorption of MB by CNC-APAM was an exothermic and spontaneous process driven by enthalpy, with a maximum adsorption capacity (*q*_m_) of 490.12 mg/g at room temperature. These findings suggest that CNC-APAM composite microspheres hold great promise as highly efficient adsorbents.

## 5. Patents

As a result of the research work elaborated in this manuscript, a patent application has been filed. The patent is titled “A Kind of Porous Polymeric Microparticles and Their Preparation Method” and was submitted to the Patent Office of the China National Intellectual Property Administration on 6 January 2025. The patent application number is CN 202510056003.5. This patent application relates to a novel preparation method, which is exactly the same as the innovative preparation method described in Section 2.2 of this paper. This preparation method represents an important achievement of this study, demonstrating the practical applications and potential value of the research. Currently, the patent is still under examination and has not been granted yet.

## Figures and Tables

**Table 1:** Effect of different CNC contents on the adsorption performance of CNC-APAM composite microspheres.

Samples	Diameter (µm)	*SR* (g·g^−1^)	*R*_e_ (%)
CNC0-APAM100	59.3	120.0 (1.8%)	86.8 (0.6%)
CNC10-APAM90	62.3	105.8 (1.5%)	92.4 (0.6%)
CNC20-APAM80	62.4	91.0 (1.3%)	93.2 (0.2%)
CNC30-APAM70	61.9	62.4 (4.8%)	93.2 (0.3%)
CNC40-APAM60	61.3	19.9 (2.5%)	93.4 (0.4%)
CNC50-APAM50	64.5	17.5 (8.6%)	93.3 (0.5%)

The value within the brackets is the relative standard deviation.

**Table 3:** Isotherm parameters for the adsorption of MB on CNC40-APAM60 microspheres at different temperatures.

Models	Parameter	Value			
		303.15 K	313.15 K	323.15 K	333.15 K
Langmuir	*q*_m_ (mg/g)	490.12	473.93	487.80	354.61
	*K*_L_ (L/mg)	0.1198	0.1040	0.0809	0.1129
	*R* ^2^	0.9985	0.9949	0.9916	0.9905
Freundlich	*K*_F_ (L/g)	165.3923	162.0379	146.3747	154.8954
	*n*	4.4324	4.6486	4.2207	6.3355
	*R* ^2^	0.9028	0.9609	0.9810	0.9159
D-R	*q*_m_ (mg/g)	472.0713	439.2022	437.7990	338.1840
	*K*_D_	0.0023	0.0020	0.0022	0.1911
	*R* ^2^	0.9821	0.9566	0.9195	0.8610
Temkin	*A* (L/g)	3.6412	3.9540	2.3030	13.4753
	*B*	32.3248	36.0253	33.8312	62.0767
	*R* ^2^	0.9407	0.9782	0.9789	0.8967

**Table 4:** Thermodynamic parameters for the adsorption of MB on CNC40-APAM60 microspheres.

*T* (K)	*K*_d_	∆*G* (kJ/mol)	∆*H* (kJ/mol)	∆
303.15	31.8816	−3.6964	−12.6456	−12.1515
313.15	32.3552	−3.8179		
323.15	29.2897	−3.9394		
333.15	19.7745	−4.0609		

**Table 5:** Comparison of the maximum adsorption capacity of different cellulose-based composite hydrogels/aerogels for MB.

Adsorbents	*q*_m_ (mg/g)	Ref.
CNC-APAM composite microspheres	490.12	This work
HPAM/CNC nanocomposite hydrogels	326.08	[26]
Graphene oxide–chitosan composite aerogels	110.9	[33]
CMC/GAs composite aerogel beads	222.72	[61]
Ti_3_C_2_TX/SA beads	92.17	[62]
CMC/ZnO/lignin hydrogel beads	276.79	[63]
Hyd/CB nanocomposite hydrogels	27.32	[64]
SPCNF aerogels	222.2	[65]
PPC/m-DE composite hydrogels	101.94	[66]
LPMCC/LPH hydrogels	57.54	[67]
CCMC/OMMT composite hydrogels	490.5	[60]

## Data Availability

The original contributions presented in the study are included in the article; further inquiries can be directed to the corresponding author.
